# Antihypertensive Medications and Change in Stages of Chronic Kidney Disease

**DOI:** 10.1155/2018/1382705

**Published:** 2018-02-25

**Authors:** Marina Komaroff, Fasika Tedla, Elizabeth Helzner, Michael A. Joseph

**Affiliations:** ^1^School of Public Health, Department of Epidemiology and Biostatistics, State University of New York Downstate Medical Center, Brooklyn, NY, USA; ^2^College of Medicine, Department of Medicine, State University of New York Downstate Medical Center, Brooklyn, NY, USA

## Abstract

**Objectives:**

The goal of this study is to estimate the change in the relationships between use of five classes of antihypertensive medications and stages of Chronic Kidney Disease (CKD) in American adults treated for hypertension.

**Methods:**

The US National Health and Nutrition Examination Survey (NHANES) data sets 1999–2012 were used with the final analytical sample of 3,045 participants. Population prevalence estimates were calculated using the NHANES survey design weights. Inferential analyses were done with binomial logistic regression models.

**Results:**

The odds of advanced (3, 4, and 5 combined) versus early CKD stages (1 and 2 combined) were significantly higher among patients treated with Angiotensin Receptor Blockers (ARB) versus those not treated with ARB in 2009–2012 (adjusted odds ratio (95% confidence interval) = 2.52 (1.32–4.80)). From 1999 to 2012, the increase in this relationship was significant (*p* = 0.0023) for users of ARB polytherapy and in users of ARB in patients with albuminuria (*p* = 0.0031).

**Conclusion:**

Aggressive pharmacological management of hypertension with ARB as add-on therapy may have accelerated kidney damage in American adults. However, prospective longitudinal studies are needed to establish proper temporal sequence in this relationship.

## 1. Introduction

Hypertension (HTN) is the second leading cause of kidney failure [1–7]. Thus, effective treatment of hypertension for American adults with CKD to slow the progression of the disease is important. In the Seventh Report of the Joint National Committee on Prevention, Detection, Evaluation, and Treatment of High Blood Pressure (JNC 7, 2003), Angiotensin Converting Enzyme (ACE) Inhibitors or Angiotensin II Receptor Blockers (ARBs) were recommended as initial or add-on pharmacological therapy particularly for patients with CKD [5, 7]. There has been a considerable increase in treatment and control of hypertension with antihypertensive medications in patients with CKD since release of JNC 7, 2003 [8–14]. Recent studies suggest a potential relationship between use of antihypertensive medications and deterioration in kidney function [15, 16]. The major point was that antihypertensive agents to control blood pressure may also impact kidney function independently of their effect on blood pressure [6].

This study examines the relationships between use of antihypertension medications and CKD stages among American adults (18 years or older) treated for hypertension and determines whether these relationships have changed from 1999 to 2012 (stacked cross-sectional design). There was public health campaign during this time period to aggressively treat and lower blood pressure particularly by use of ACE I and ARBs in patients with CKD. We hypothesized that if the recommended treatment of hypertension in patients with CKD worked as intended then the associations with stages of CKD should be moving towards a decrease in the prevalence of advanced stages or at least stay stable; otherwise, the recommended therapy might appear to be more harmful than beneficial and should perhaps be revised.

## 2. Material and Methods

### 2.1. Database Overview

The US National Health and Nutrition Examination Survey (NHANES) is a representative sample of noninstitutionalized US civilian residents that are currently conducted every 2 years. NHANES data are obtained using a complex, multistage, cluster, and probability sampling design that incorporates differential probabilities of selecting participants. The data collection, reporting, and analyses followed consistent definitions that can be found on the websites [17].

### 2.2. Study Design

Three time points (NHANES cycles 1999–2004, 2005–2008, and 2009–2012) were utilized to determine if these relationships have changed over time (stacked cross-sectional design).

### 2.3. Study Population

Inclusion criteria were participants 18 years of age or older, who completed questionnaire and underwent a physical examination, reported demographic information (age, gender, and race), provided self-reported information on hypertension including treatment, had blood pressure measured, had estimated glomerular filtration rate (eGFR) and urine albumin/creatinine ratio (ACR), and were not pregnant if female. Population of interest was participants with hypertensive CKD, who satisfied inclusion criteria, and were treated for hypertension.

### 2.4. Definitions

CKD was defined by the level of glomerular filtration rate (GFR) and the urine albumin-to-creatinine ratio (ACR) [6, 18]. The stages of CKD and albumin to creatinine ratio (ACR) were classified following the National Kidney Foundation (NKF) Kidney Disease Outcomes Quality Initiative (KDOQI) [6, 18].

Hypertension for participants with CKD was present if systolic blood pressure is greater than or equal to 130 mm Hg or diastolic blood pressure greater than or equal to 80 mm Hg, or subject currently was taking medication to lower high blood pressure [7, 19].

Treatment of high blood pressure was defined by an affirmative response to the question “Have you ever been told by a doctor or health professional that you had hypertension, also called high blood pressure?” and to the following questions: “Because of your high blood pressure/hypertension, have you ever been told to take prescribed medicine?” and “Are you now following this advice to take prescribed medicine?” [7, 19].

Diabetes was present if glycohemoglobin (HbA1c) was equal to or greater than 6.5% [20].

The five antihypertensive classes of medications were (1) ACE I (Angiotensin Converting Enzyme Inhibitors); (2) ARB (Angiotensin Receptor Blockers); (3) BB (*β*-blockers); (4) CCB (Calcium Channel Blockers); and (5) Diuretics [5, 7]. Monotherapy was defined when participates reported use of only one antihypertensive drug, and polytherapy which is the same as combination therapy was defined when participants reported use of more than one antihypertensive agent. When single pill of antihypertensive combination was reported, it was classified as polytherapy.

**Figure 1:**
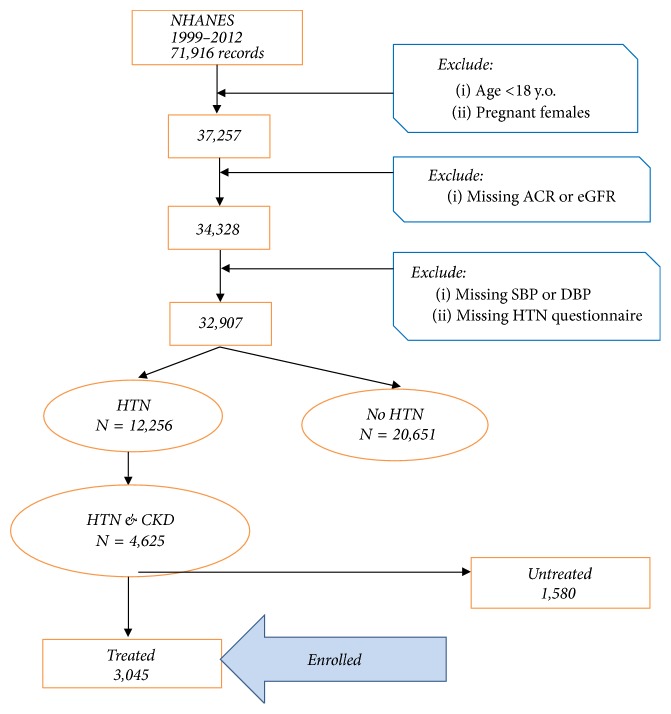
Consort flow diagram.

Data from seven NHANES surveys 1999–2012 included 71,916 records. The analytical sample of hypertensive participants with CKD who were treated for HTN and satisfied inclusion criteria was 3,045; see consort flow diagram, Figure 1.

### 2.5. Statistical Analyses

The prevalence of CKD by classes of antihypertensive medications was age-standardized to the 2000 US standard population using 4 age groups [19, 21]. Variance estimation was performed utilizing Taylor series method. Relative standard errors (RSE) were calculated, and those greater than 30% were considered potentially statistically unreliable per NHANES Analytic Guidance and marked with the asterisk (*∗*) in the tables [21]. Because of small sample size of severe advanced stages (4 or 5), the main analyses were focused on CKD early stages 1 and 2 combined and advanced stages 3, 4, and 5 combined.

#### 2.5.1. Model Description

Binomial logistic regression models were used to test the hypotheses of the study. In the models for three time points (NHANES cycles 1999–2004, 2005–2008, and 2009–2012), the ordinal coded dichotomous variable for stages of CKD was the dependent variable (outcome), time was independent variable, and class of antihypertensive medication was the independent variable (effect modifier). Interaction terms (time × class of medication) or (time × mono- or polytherapy) were used to test the null hypothesis of no difference in change in the odds ratios for patients treated versus untreated with particular class of medication over 14-year time period. The models included the following covariates: age, gender, race/ethnicity, body mass index (BMI), smoking status, diabetes, and albuminuria to adjust for potential confounding. The models accommodated the complex multistage sample survey design following NHANES Analytic and Reporting guidance [21]. To preserve Type I error in multiple subgroup analyses, the Bonferroni correction *α*/*k* was used where k is the number of analyses.

#### 2.5.2. Trend Analyses

Trend was determined with the statistically significant interaction between class of medication and time (ordinal variable) indicating that their relationships had changed over time. In the models, the outcome variable was modeled as Logits that contrasted advanced (3, 4, and 5 combined) with the earlier (1 and 2 combined) stages of CKD. The odds ratios (95% Confidence Interval) were reported for each of five classes of medications: ACEI, ARB, BB, CCB, DIU, and poly- or monotherapy.

*Exploratory Analyses*. Subgroup analyses were performed with the same model and covariates for the following groups: diabetes (absent/present); albuminuria/ACR >30 mg/g (absent/present); microalbuminuria/ACR 30–<300 mg/g (absent/present); macroalbuminuria/ACR >300 mg/g (absent/present); diabetes with albuminuria; diabetes with microalbuminuria; diabetes with macroalbuminuria; diabetes without albuminuria; diabetes without microalbuminuria; diabetes without macroalbuminuria; no diabetes and albuminuria; no diabetes and microalbuminuria; no diabetes and macroalbuminuria.

Statistical analyses were conducted using the SAS® System for Windows (release 9.3; SAS® Institute Inc., Cary, NC) and utilized the SURVEY procedures that account for NHANES survey multistage complex design [21].

## 3. Results

The use of medications other than the five major classes decreased from about 6.5% in 1999–2004 to 4.6% in 2005–2008 and 3.9% in 2009–2012. Those “other” included miscellaneous cardiovascular agents, vasodilators, vasopressors, agents for pulmonary hypertension, aldosterone receptor antagonists, and renin inhibitors; and they were not considered in this study. The use of alpha-1 blockers increased from 4.27% in 1999–2004 to 5.44% in 2005–2008 and 6% in 2009–2012. Similarly, the use of central alpha-2 antagonists and other centrally acting antiadrenergic agents increased from 3.58% in 1999–2004 to 3.81% in 2005–2008 and 5.28% in 2009–2012. This study did not examine alpha-blockers, but focused only on the use of five major classes of antihypertensive medications recommended by JNC-7 to treat hypertension in patients with CKD [7].

### 3.1. Prevalence of Use of Antihypertensive Medications

From 1999 to 2012, the prevalence % (SE) of adults with CKD and HTN who were treated for HTN increased from 55.58% (1.81) to 64.76% (1.91). Among five classes of medications, the use of ARB polytherapy increased the most: from 12.26% (1.44) to 21.56% (2.22); and the use of DIU polytherapy decreased the most: from 49% (2.55) to 42.05% (2.35); from 1999–2004 to 2009–2012. Two out of three patients with CKD and HTN received combination of classes (Table 1).

The prevalence of users of more than one class decreased from 65.76% (2.24) to 62.21% (3.31); however, the distributions by CKD stages for these users demonstrated increase in advanced stages, from 55.45% (2.28) to 56.20% (3.59) in stage 3 and from 7.33% (1.62) to 8.97% (1.85) in stages 4 and 5 combined, and decrease in early stages (1 and 2) from 37.21% to 34.82% (Table 1). The prevalence of patients was consistently greater in advanced (3) versus any early stages (1 or 2) among uses of each class. However, the increase in advanced stages was observed for ARB polytherapy and Diuretics monotherapy. The most increase in advanced stages was observed for ARB polytherapy: from 49.97% (5.61) to 70.72% (5.06) for stage 3 and from 5.7% (1.96) to 7.42% (1.94) for stages 4 and 5; while the early stages decreased from 29.58% (6.57) to 15.87% (4.38) for stage 2 and from 14.74% (5.72) to 5.98% (2.48) for stage 2.

### 3.2. Antihypertensive Medications and CKD Stages: Trend Analyses

**Figure 2:**
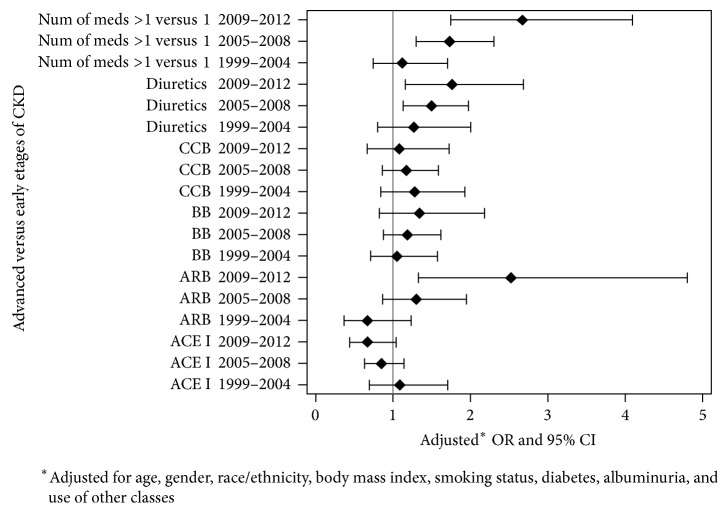
Forest-plots of adjusted^*∗*^ odds ratios (95% CI) for advanced (3, 4, and 5) versus early (1 and 2) stages of CKD by use of five classes of medication.* Note*. ACEI = Angiotensin Converting Enzyme Inhibitors; ARB = Angiotensin Receptor Blockers; BB = *β*-blockers; CCB = Calcium Channel Blockers; DIU = Diuretics.

The significant increase in the odds of advanced versus early stages was observed for ARB users (*p* = 0.0069), users of ARB polytherapy (*p* = 0.0023), and for users of poly- versus monotherapy (*p* = 0.0063); see Table 2 and Figure 2.  Table 3 summarizes the prevalence of combinations of five antihypertensive classes of medication. The least prevalent was combination of ARB with ACEI therapy. Moreover, combinations of ARB with ACEI demonstrated decreased prevalence from 2005 through 2012: from 0.33% (SE = 0.18) to 0.04% (SE = 0.03) for ARB + ACEI; 0.57% (SE = 0.51) to 0.14% (SE = 0.11) for ARB + ACEI + BB; 0.22% (SE = 0.20) to 0.06% (SE = 0.04) for ARB + ACEI + CCB; 0.21% (SE = 0.12) to 0.03% (SE = 0.03) for ARB + ACEI + DIU; 0.33% (SE = 0.21) to 0.03% (SE = 0.03) for ARB + ACEI + BB + CCB; and 0.21% (SE = 0.15) to 0.17% (SE = 0.11) for ARB + ACEI + BB + DIU. Increased prevalence of medication combinations was observed only for ARB + ACEI + CCB + DIU from 0.08% (SE = 0.06) to 0.14% (SE = 0.08) and for ARB + ACEI + CCB + DIU + BB from 0.17% (SE = 0.10) to 0.49% (0.24) from 2005 through 2012. The most prevalent combinations with ARB were ARB + DIU and ARB + DIU + BB with the prevalence above 4% from 2005 through 2012. During the same period of time, the use of combinations with ARB increased more than 2% for ARB + BB + CCB: from 0.40% (SE = 0.14) to 2.29% (SE = 0.88). The other combination with ARB was above 1% and increased from 1.11% (SE = 0.21) to 1.92% (SE=0.76) for ARB + BB, from 1.81% (SE = 0.61) to 2.88% (SE = 0.81) for ARB + CCB + DIU, and from 1.67% (SE = 0.61) to 1.88% (SE = 0.52) for ARB + BB + CCB + DIU and slightly decreased from 1.88% (SE=0.64) to 1.82% (SE = 1.12) for ARB + CCB. None of the combinations of classes of medications with ARB demonstrated significant change in the relationships between advanced versus earlier stages of CKD from 1999 through 2012, except ARB + CCB (*p* = 0.0065). However, complete analysis cannot be performed, particularly for ARB combinations with ACEI due to small sample sizes.

The exploratory analyses were conducted with level of significance *α*/15 = 0.0033 (Bonferroni correction for *α* = 0.05) where 15 is the number of analyses. The same models stratified by subgroups demonstrated significant increase in odds of advanced versus early stages of CKD for: (1) ARB users among patients with albuminuria (*p* = 0.0031) and (2) users of more than one class of medications with albuminuria (*p* = 0.002); table is not present.

## 4. Discussion

The major finding is that there was significant increase in this relationship for the users of ARB polytherapy (*p* = 0.0023) from 1999 through 2012. The trend can be explained by the increase in prevalence of advanced stages CKD and decrease or stable prevalence in early stages among users of ARB polytherapy. Such increase in advanced CKD stages may have been explained by use of ARB combinations with ACEI that was particularly warned by JNC-7, but those combinations were least prevalent and decreased during the investigated time period. Possibly, change in the relationship was driven by ARB combinations with other classes, particularly with CCB where odds of advanced stages significantly increased compared to the odds of earlier stages, from 1999 through 2012. A significant increase in advanced stages was observed for ARB users among people with albuminuria. The results suggest that increase in ARB polytherapy may have increased advanced stages of CKD among American adults, particularly among people with albuminuria.

In this study for American adults 18 years or older with CKD, the changes in age-standardized prevalence were from 55.58% to 64.76%, from 1999–2004 to 2009–2012 (Table 1). Gu et al. reported similar significant (*p* = 0.03) increase in unadjusted prevalence of users of multiple antihypertensive drugs in this population of adults with CKD from 54.2% (3.5) to 65.6% (3.0), from 2001-2002 to 2009-2010 [22]. But the prevalence by five classes of medications reported by Gu et al. was smaller to that reported in this study that can be explained by having population of adults with CKD as comorbid condition in this study. For example, in the Gu et al. study the change in prevalence of ARB users went from 10.5% (1.1) to 22.2% (1.6) for poly- or monotherapy, from 7.6% (0.9) to 16.1% (1.1) for polytherapy, and from 3.0% (0.4) to 4.9% (0.7) for monotherapy: NHANES from 2001-2002 to 2009-2010 [22]. In the present study, the increase in ARB users among hypertensive adults with CKD went from 15.93% (1.78) to 26.1% (2.37) for poly- or monotherapy, from 12.26% (1.44) to 21.56% (2.22) for polytherapy, and from 3.67% (0.99) to 4.54% (0.74) for monotherapy: NHANES from 1999–2004 to 2009–2012. The prevalence of advanced stages for the users of ARBs polytherapy increased from about 49.97% (5.61) to 70.72% (5.06) in stage 3 and from 5.70% (1.96) to 7.42% (1.94) in stages 4 and 5 combined, while the early stages decreased about half as much. The strength of the association suggests that the aggressive treatment of patients with CKD by ARB could be responsible for kidney damage and the increase in advanced stages of CKD. A first population-based ecological study in the United Kingdom demonstrated an association between the increase in hospital admission rates for acute kidney injury (AKI) and the increase in prescriptions of antihypertensive medications (ACE inhibitors/ARA) in medical practices over a 4-year time period [15]. The authors concluded that 15% of the increase in AKI admission was potentially attributable to increased prescriptions of ACE I and ARBs [15].

The current study is the first study to the authors' knowledge that estimated changes in relationships between use of antihypertensive medications and stages of CKD for American hypertensive adults with CKD. The results are consistent with the population-based ecological study in the United Kingdom and suggest that significant increase in advanced stages of CKD can be potentially attributable to the treatment with ARB polytherapy, perhaps, damaging the kidney and increasing albuminuria. However, the temporal relationship between ARB therapy and increase in advanced stages of CKD cannot be established because of the cross-sectional nature of this study. It is possible that increase in ARB therapy resulted from the increase in advanced stages of CKD. Prospective longitudinal studies are needed to establish proper temporal sequence of this relationship.

### 4.1. Limitations and Strengths

The major limitation is that cross-sectional NHANES data does not allow assessing the relationship between CKD and treatment of hypertension longitudinally within patients. Another limitation is that blood pressure was measured on a single day versus on more than two separate days as was recommended by JNC 7 guidance [7]. However, three measurements were taken that might have minimized this misclassification. Similarly, CKD was defined based on single laboratory measurements versus multiple measurements during more than three months defined by KDOQI guidance [18]. However, the bias is minimal for the estimates that were the goal of this study. NHANES is a representative sample of the noninstitutionalized US population; yet, older people or people with severe cases, such as stage 3 or 4 of CKD, might have been underreported because usually weaker populations were less likely to attend MEC examination. Combining the advanced versus early stages in the statistical analyses minimized the population selection bias. Small sample size per each stage of CKD did not allow separate analysis by each stage. The combinations of classes of antihypertensive medications cannot be fully examined due to small sample sizes. Lastly, although the relationships between antihypertension medications and CKD stages are intriguing, NHANES data does not permit analysis of prescribing practices of physicians. The strength of the study is the use of NHANES data with a large sample size, good quality control, and comprehensive data collection. The long period of consistent data collected gives change to investigate the trend in the relationships between CKD stages and treatment of HTN before and after publishing JNC-7 guidelines. The presence of major risk factors for CKD and HTN was also consistently collected that gives us confidence in the results.

## 5. Conclusions

The link between ARBs and acute kidney injures was recognized recently by KDIOGO and UK National Institute for Health and Clinical Excellence (NICE) [23, 24]. The results from this study suggest that increase in treatment with ARB possibly increased the prevalence of advanced CKD stages among American adults by damaging the kidneys. However, the temporal relationship cannot be claimed from this cross-sectional study. Prospective studies will be necessary to establish proper temporal sequence.

## Figures and Tables

**Table 1:** Age-standardized^$^ prevalence of American adults (18 years of age or older) with hypertensive CKD treated for hypertension by five classes of medications and CKD stages: NHANES 1999–2012.

Population	CKD	1999–2004 % (SE)	2005–2008 % (SE)	2009–2012 % (SE)
*CKD & HTN treated*	Total	*N* = 1122 55.58 (1.81)	*N* = 916 62.13 (1.87)	*N* = 1007 64.76 (1.91)
	Stage 1	17.80 (2.15)	17.18 (1.83)	16.65 (2.01)
	Stage 2	24.81 (2.43)	26.64 (2.36)	25.06 (2.68)
	Stage 3	51.38 (2.68)	52.08 (3.40)	52.10 (3.19)
	Stages 4 & 5	6.00 (1.34)	4.09 (0.78)	6.17 (1.02)

*Poly- or monotherapy*		*N* = 1049	*N* = 874	*N* = 968
Angiotensin Converting Enzyme Inhibitors (ACE I)	Total	462	395	424
		42.48 (1.93)	47.68 (1.68)	40.40 (2.08)
	Stage 1	13.91 (2.98)	17.38 (2.58)	20.49 (3.22)
	Stage 2	28.61 (4.46)	31.59 (3.58)	28.40 (3.38)
	Stage 3	51.40 (3.89)	48.42 (3.89)	46.14 (4.03)
	Stages 4 & 5	6.08^∗^ (1.89)	2.60 (0.70)	4.96^∗^ (1.79)
Angiotensin Receptor Blockers (ARB)	Total	176	218	282
		15.93 (1.78)	22.30 (2.30)	26.10 (2.37)
	Stage 1	18.28 (4.76)	12.13^∗^ (4.04)	5.83^∗^ (2.24)
	Stage 2	24.88 (5.42)	22.72 (5.47)	19.56 (5.32)
	Stage 3	52.17 (5.07)	59.24 (6.43)	66.56 (5.16)
	Stages 4 & 5	4.65^∗^ (1.50)	5.89^∗^ (2.08)	8.03 (1.89)
*β*-blockers (BB)	Total	365	434	465
		38.14 (2.35)	43.62 (2.70)	41.64 (3.16)
	Stage 1	13.07 (3.13)	14.41 (3.76)	9.29 (2.27)
	Stage 2	23.04 (3.02)	24.95 (3.51)	26.90 (3.84)
	Stage 3	55.20 (3.58)	53.36 (4.96)	54.38 (4.00)
	Stages 4 & 5	8.69 (2.12)	7.27 (1.58)	9.41 (2.16)
Calcium Channel Blockers (CCB)	Total	427	332	339
		32.39 (1.92)	32.84 (2.17)	30.34 (3.21)
	Stage 1	14.69 (3.18)	16.46 (3.64)	20.29 (4.08)
	Stage 2	26.76 (4.21)	25.41 (4.15)	21.51 (3.89)
	Stage 3	47.83 (3.82)	53.15 (5.71)	48.53 (4.69)
	Stages 4 & 5	10.71 (2.89)	4.98 (1.47)	9.66 (2.14)
Diuretics (DIU)	Total	549	495	494
		51.95 (2.68)	51.06 (2.50)	47.77 (2.35)
	Stage 1	15.21 (2.53)	13.86 (2.87)	15.34 (2.90)
	Stage 2	22.19 (3.10)	26.82 (3.53)	18.80 (2.73)
	Stage 3	58.07 (3.27)	54.33 (4.04)	58.62 (3.94)
	Stages 4 & 5	4.51 (1.10)	4.98 (1.26)	7.23 (1.60)

*Polytherapy*				
Angiotensin Converting Enzyme Inhibitors (ACE I)	Total	345	317	324
		31.10 (1.62)	34.68 (2.19)	28.81 (2.42)
	Stage 1	11.49 (2.45)	14.85 (2.42)	18.46 (3.73)
	Stage 2	23.99 (4.32)	30.01 (3.47)	27.24 (4.25)
	Stage 3	55.36 (4.21)	51.72 (4.40)	48.53 (3.86)
	Stages 4 & 5	9.16^∗^ (3.07)	3.41 (1.01)	5.76^∗^ (2.03)
Angiotensin Receptor Blockers (ARB)	Total	144	189	227
		12.26 (1.44)	18.17 (2.21)	21.56 (2.22)
	Stage 1	14.74^∗^ (5.72)	14.58^∗^ (4.86)	5.98^∗^ (2.48)
	Stage 2	29.58 (6.57)	24.02 (6.03)	15.87 (4.38)
	Stage 3	49.97 (5.61)	55.15 (6.53)	70.72 (5.06)
	Stages 4 & 5	5.70^∗^ (1.96)	6.24^∗^ (1.87)	7.42 (1.94)
*β*-blockers (BB)	Total	315	370	404
		32.45 (2.06)	36.08 (2.39)	34.33 (2.71)
	Stage 1	10.92 (2.93)	12.26^∗^ (3.91)	11.50 (2.94)
	Stage 2	23.47 (3.37)	25.75 (3.65)	20.72 (3.90)
	Stage 3	56.55 (3.65)	54.99 (5.21)	54.89 (4.35)
	Stages 4 & 5	9.06 (2.17)	6.99 (1.78)	12.87 (3.28)
Calcium Channel Blockers (CCB)	Total	309	298	285
		24.72 (1.91)	29.81 (2.22)	24.64 (3.10)
	Stage 1	9.88 (2.55)	14.00 (3.41)	18.49 (4.37)
	Stage 2	26.28 (4.79)	25.47 (4.38)	19.55 (4.24)
	Stage 3	50.59 (4.29)	55.01 (6.18)	50.64 (4.80)
	Stages 4 & 5	13.24 (3.46)	5.51 (1.60)	11.31 (2.61)
Diuretics (DIU)	Total	511	470	460
		49.36 (2.55)	46.98 (2.34)	42.05 (2.35)
	Stage 1	14.76 (2.57)	15.68 (3.51)	12.04 (3.20)
	Stage 2	22.09 (3.19)	26.32 (3.98)	19.00 (3.14)
	Stage 3	58.61 (3.31)	52.71 (4.21)	59.86 (4.48)
	Stages 4 & 5	4.53 (1.16)	5.28 (1.34)	9.09 (2.43)

*Monotherapy*				
Angiotensin Converting Enzyme Inhibitors (ACE I)	Total	117	78	100
		11.38 (1.59)	13.00 (2.18)	11.59 (1.78)
	Stage 1	16.63^∗^ (5.53)	21.82 (4.47)	24.97 (5.16)
	Stage 2	36.24 (6.89)	33.29 (5.41)	32.19 (6.85)
	Stage 3	45.40 (5.76)	44.49 (5.70)	41.60 (6.72)
	Stages 4 & 5	1.72^∗^ (1.19)	0.39^∗^ (0.41)	1.24^∗^ (0.65)
Angiotensin Receptor Blockers (ARB)	Total	32	29	55
		3.67 (0.99)	4.13 (1.08)	4.54 (0.74)
	Stage 1	27.57^∗^ (9.96)		4.85^∗^ (3.12)
	Stage 2	9.41^∗^ (4.24)		37.07^∗^ (12.63)
	Stage 3	62.22 (10.37)		47.57 (8.88)
	Stages 4 & 5	0.79^∗^ (0.81)		10.50^∗^ (5.34)
*β*-blockers (BB)	Total	50	64	61
		5.69 (1.36)	7.55 (1.42)	7.31 (1.75)
	Stage 1		25.90 (6.64)	1.43^∗^ (1.18)
	Stage 2		22.04^∗^ (7.71)	43.74 (10.40)
	Stage 3		44.35 (9.61)	54.53 (10.46)
	Stages 4 & 5		7.69^∗^ (4.15)	0.29^∗^ (0.29)
Calcium Channel Blockers (CCB)	Total	118	34	54
		7.67 (1.21)	3.03 (0.71)	5.70 (1.29)
	Stage 1	28.52 (5.62)		27.20 (7.21)
	Stage 2	28.85 (6.87)		30.38 (7.91)
	Stage 3	39.36 (5.46)		39.04 (10.77)
	Stages 4 & 5	3.26^∗^ (1.28)		3.38^∗^ (2.04)
Diuretics (DIU)	Total	38	25	34
		2.59 (0.62)	4.08^∗^ (1.34)	5.73 (1.65)
	Stage 1	20.99^∗^ (8.61)	6.71^∗^ (4.92)	29.59 (6.83)
	Stage 2	29.29^∗^ (11.04)	19.98^∗^ (8.86)	11.98^∗^ (5.43)
	Stage 3	45.03 (11.72)	73.31 (8.34)	58.41 (7.14)
	Stages 4 & 5	4.69^∗^ (3.15)	0	0

*Monotherapy* *Number of class meds = 1*	Total	382	241	312
		31.69 (2.18)	32.51 (2.68)	35.24 (2.94)
	Stage 1	22.71 (3.83)	20.64 (2.06)	19.11 (3.37)
	Stage 2	28.15 (4.43)	26.10 (4.31)	32.31 (3.75)
	Stage 3	46.37 (3.59)	50.82 (4.68)	46.21 (4.63)
	Stages 4 & 5	2.76 (0.83)	2.43^∗^ (0.97)	2.35^∗^ (0.81)

*Polytherapy* *Number of class meds > 1*	Total	667	633	656
		65.76 (2.24)	66.32 (2.71)	62.21 (3.31)
	Stage 1	13.93 (2.49)	14.35 (2.43)	14.65 (2.94)
	Stage 2	23.28 (2.86)	26.65 (2.80)	20.17 (2.86)
	Stage 3	55.45 (2.82)	53.97 (4.13)	56.20 (3.59)
	Stages 4 & 5	7.33 (1.62)	5.03 (1.10)	8.97 (1.85)

^*∗*^Potentially statistically unreliable estimates with relative standard errors > 30%. ^$^Age-standardized to the 2000 US standard population using 4 age groups.

**Table 2:** Adjusted^##^ odds ratios (95% CI) for advanced (3, 4, and 5) versus early (1 and 2) stages of CKD by use of each class of medication.

Model: class med^$^	1999–2004 odds ratio (95% CI)	2005–2008 odds ratio (95% CI)	2009–2012 odds ratio (95% CI)	*p* ^#^
*ACE I*				
Overall	1.08 (0.69–1.70)	0.85 (0.63–1.14)	0.67 (0.43–1.04)	0.1574
Polytherapy	0.93 (0.57–1.52)	0.83 (0.60–1.15)	0.74 (0.46–1.20)	0.5417
Monotherapy	1.59 (0.91–2.80)	1.06 (0.65–1.73)	0.70 (0.33–1.48)	0.0713
*ARB*				
Overall	0.67 (0.36–1.23)	1.30 (0.87–1.94)	2.52 (1.32–4.80)	**0.0069**
Polytherapy	0.55 (0.29–1.03)	1.20 (0.80–1.80)	2.63 (1.37–5.07)	**0.0023**
Monotherapy	1.97 (0.63–6.18)	1.66 (0.83–3.31)	1.39 (0.56–3.46)	0.6575
*BB*				
Overall	1.05 (0.71–1.57)	1.19 (0.87–1.61)	1.34 (0.82–2.17)	0.4637
Polytherapy	1.05 (0.67–1.65)	1.23 (0.89–1.69)	1.43 (0.91–2.27)	0.3490
Monotherapy	0.98 (0.43–2.28)	0.88 (0.47–1.64)	0.78 (0.29–2.13)	0.7414
*CCB*				
Overall	1.28 (0.85–1.93)	1.17 (0.86–1.59)	1.08 (0.67–1.72)	0.5953
Polytherapy	1.14 (0.73–1.78)	1.22 (0.88–1.70)	1.31 (0.77–2.22)	0.7036
Monotherapy	1.24 (0.63–2.46)	0.78 (0.45–1.36)	0.49 (0.24–1.02)	**0.0359**
*DIU*				
Overall	1.27 (0.81–2.00)	1.49 (1.13–1.98)	1.76 (1.16–2.67)	0.3344
Polytherapy	1.37 (0.88–2.13)	1.65 (1.24–2.20)	1.99 (1.29–3.06)	0.2665
Monotherapy	0.31 (0.11–0.91)	0.30 (0.12–0.73)	0.29 (0.06–1.29)	0.9288
*Number of Classes med >1 (polytherapy) versus = 1 (monotherapy)*				
Combination	1.12 (0.74–1.70)	1.73 (1.30–2.30)	2.67 (1.74–4.09)	**0.0063**

^$^ACEI = Angiotensin Converting Enzyme Inhibitors; ARB = Angiotensin Receptor Blockers; BB = *β*-blockers; CCB = Calcium Channel Blockers; DIU = Diuretics; ^#^PROC SURVEYLOGISTIC was used to test the trend; ^##^Adjusted for: age, gender, race, body mass index, smoking status diabetes, and albuminuria; *NOTE*. Significant associations and *p*-values with level of significance *α* = 0.05 are presented *in bold*.

**Table 3:** Age-standardized prevalence of American adults (18 years of age or older) with hypertensive CKD treated For hypertension by combination of five classes of medications: NHANES 1999–2012.

Combination of classes	1999–2004 % (SE)	2005–2008 % (SE)	2009–2012 % (SE)
ACE I + ARB	0.12^∗^ (0.09)	0.33^∗^ (0.18)	0.04^∗^ (0.03)
ACE I + BB	3.41 (0.89)	4.06 (1.02)	4.24 (0.73)
ACE I + CCB	4.59 (0.86)	4.39 (0.88)	3.24^∗^ (1.22)
ACE I + DIU	10.82 (1.54)	6.84 (1.17)	7.36 (1.33)
ARB + BB	0.52^∗^ (0.19)	1.11 (0.21)	1.92^∗^ (0.76)
ARB + CCB^$^	0.44^∗^ (0.16)	1.88^∗^ (0.64)	1.82^∗^ (1.12)
ARB + DIU	4.22 (0.91)	4.31 (1.05)	4.64 (0.85)
BB + CCB	2.12 (0.51)	2.88 (0.64)	1.44 (0.41)
BB + DIUX	12.11 (1.72)	5.48 (0.92)	5.21 (1.37)
CCB + DIUX	3.85 (0.69)	3.28 (0.96)	1.91^∗^ (0.82)
ACE I + ARB + BB	0.00 (0.00)	0.57^∗^ (0.51)	0.14^∗^ (0.11)
ACE I + ARB + CCB	0.22^∗^ (0.16)	0.22^∗^ (0.20)	0.06^∗^ (0.04)
ACE I + ARB + DIU	0.17^∗^ (0.14)	0.21^∗^ (0.12)	0.03^∗^ (0.03)
ACE I + BB + CCB	2.45^∗^ (0.91)	2.40^∗^ (0.96)	2.00 (0.48)
ACE I + BB + DIU	2.23 (0.50)	6.79 (1.22)	5.74 (1.13)
ACE I + CCB + DIU	3.09 (0.70)	5.02 (1.30)	2.39^∗^ (0.82)
ARB + BB + CCB	0.34^∗^ (0.26)	0.40^∗^ (0.14)	2.49^∗^ (0.88)
ARB + BB + DIU	3.08 (0.69)	4.70 (0.81)	4.66 (1.27)
ARB + CCB + DIU	1.57^∗^ (0.50)	1.81^∗^ (0.61)	2.88 (0.81)
BB + CCB + DIU	2.13 (0.55)	2.25 (0.52)	1.11^∗^ (0.35)
ACE I + ARB + BB + CCB	0.04^∗^ (0.04)	0.33^∗^ (0.21)	0.03^∗^ (0.03)
ACE I + ARB + BB + DIU	0.35^∗^ (0.19)	0.21^∗^ (0.15)	0.17^∗^ (0.11)
ACE I + ARB + CCB + DIU	0.48^∗^ (0.48)	0.08^∗^ (0.06)	0.14^∗^ (0.08)
ARB + BB + CCB + DIU	0.31^∗^ (0.12)	1.67^∗^ (0.61)	1.88 (0.52)
ACEI + ARB + BB + CCB + DIU	0.20^∗^ (0.16)	0.17^∗^ (0.10)	0.49^∗^ (0.24)

ACEI = Angiotensin Converting Enzyme Inhibitors; ARB = Angiotensin Receptor Blockers; BB = *β*-blockers; CCB = Calcium Channel Blockers; DIU = Diuretics. ^∗^Potentially statistically unreliable estimates with relative standard errors > 30%. ^$^The use of this combination demonstrated significant increase in odds of advanced stages of CKD compared to odd of early stages, 1999 through 2012 (*p* = 0.0065).
